# Letter to the editor for the article “Risk factors for pulmonary complications after colorectal cancer surgery: a Japanese multicenter study”

**DOI:** 10.1007/s00384-024-04667-y

**Published:** 2024-06-21

**Authors:** Ya-jie Wang, Yi-sheng Pan

**Affiliations:** Department of Gastrointestinal Surgery, Peking University First Hospital, No.8 Xishku Street, Xicheng District, Beijing, China

## Dear Editor:

We were interested to read the impressive study “Risk factors for pulmonary complications after colorectal cancer surgery: a Japanese multicenter study” by Tetsuro Tominaga et al. in ***International Journal of Colorectal Disease*** [1] and would like to congratulate the authors for their interesting findings. The study focusing on the identification of influential factors contributing to postoperative pulmonary complications in patients undergoing colorectal cancer surgery is a significant contribution to understanding the multifactorial nature of such adverse outcomes. Through thorough analysis across several Japanese centers, the investigation highlights critical insights into these complications, notably demonstrating an association with male gender, older age, underweight conditions, and poorer ASA-PS scores. Nonetheless, it is worth considering certain methodological points for a more detailed exploration of this topic.

For the one thing, in the approach of including variables with a *p* value of less than 0.05 in univariate analysis into the multivariate analysis is a widely accepted method. However, it is noteworthy that the univariate analysis indicating the association of male gender with a *p* value of 0.09 (odds ratio was 1.634) was still included in the multivariate analysis. That might be inconsistent. Although the exclusion of variables based solely on *p* values from univariate analysis can be overly restrictive, a cautious approach to the inclusion of variables with borderline significance may warrant some reconsideration in order to enhance the robustness of the analysis.

Furthermore, we believe that the study would significantly benefit from exploring the effects of smoking habits on postoperative pulmonary complications [2]. Given the well-established impacts of smoking on lung function and the possible amplification of these effects following surgical procedures, it would be informative to include data on smoking status as an additional layer of analysis.

In addition, regarding ASA-PS scores, it is prudent to clarify whether Table 3 indicates the presence of complications in patients with ASA-PS scores of 3 or greater. A clear description of the scoring thresholds within the context of complications would provide a clearer picture of the relationship between ASA-PS scores and pulmonary complications [3].

Finally, the authors’ efforts in highlighting significant variables influencing postoperative pulmonary complications in colorectal cancer patients are laudable and provide substantial contributions to the field. The insights gained from this study could inform future research and the refinement of clinical practices aimed at mitigating such complications. We are grateful for the thoughtful and systematic evaluation provided in this work and look forward to further contributions that build upon these findings.

## Data Availability

Not applicable.
